# Using archaeological data for the understanding of Late-Holocene Sea of Galilee’s level fluctuations

**DOI:** 10.1038/s41598-022-09768-8

**Published:** 2022-06-13

**Authors:** Matthieu Giaime, Michal Artzy

**Affiliations:** 1 Institut de Ciència i Tecnologia Ambientals (ICTA-UAB), Universitat Autònoma de Barcelona, 08193 Cerdanyola del Vallès, Barcelona, Spain; 2 Hatter Laboratory, Recanati Institute for Maritime Studies, Department of Maritime Civilizations, University of Haifa, 3498838 Haifa, Israel

**Keywords:** Climate-change adaptation, Climate-change impacts, Environmental impact

## Abstract

In the Jordan Valley, reconstructed changes of the Sea of Galilee level have shown sharp fluctuations of the water elevation during the Holocene. In this paper, we provide new data originating from the excavations of Kursi Beach archaeological site located on the eastern shore of the Sea of Galilee and compare them with other data gathered from the archaeological site of Magdala, located on its western shore. Our data yield to constrain Sea of Galilee level changes between the Iron Age II (10th–9th centuries BCE) and the Crusader period (11th–12th centuries CE), a period of high interest for the archaeological community. We demonstrate that water level was around -212 to -210 m mean sea level (msl) for the Iron Age II period. Lake level rose to -208/-209 m msl during the Late Hellenistic/Early Roman period. Water level remained low (<-213/-214 m msl) from the Byzantine to the Crusader period (from 5th to 12th centuries CE). Our data provide new knowledge for the understanding of variations in the Sea of Galilee level in antiquity. We highlight that water level fluctuations must have been key factors taken into account in the habitation pattern.

## Introduction

**Figure 1:**
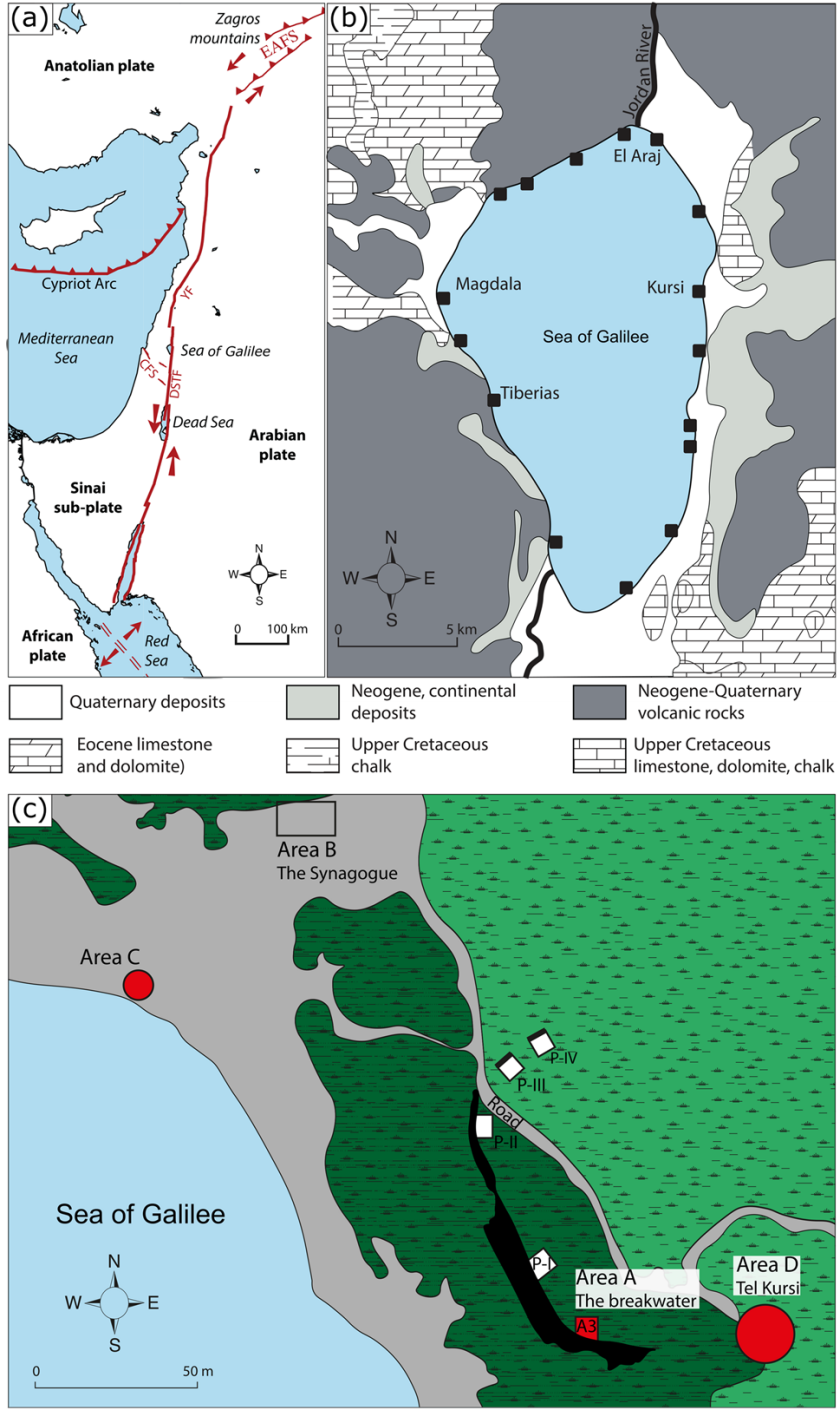
Geographical context. (**a**) Simplified tectonic map of the eastern Mediterranean, showing the plate boundaries and the main faults^14^. DSTF, Dead Sea Transform; CFS, Carmel Fault System; YF, Yammouneh Fault. (**b**) Geological map of the Sea of Galilee^15^. The black squares indicate the main built harbors on the shore of the lake^16^. (**c**) Kursi Beach general plan of the excavation. Original measurements and drawing by M. Edelcopp and B. Arubas.

The Jordan Valley was a cradle of human occupation and saw the early development of complex societies^1, 2^. Along the valley, freshwater lakes and marshes attracted local inhabitants to their shorelines and they, in turn, closely interacted with their natural environment^3, 4^. These anthropogenic legacies, modified over the millennia constitute the cultural heritage of the region. The strong interest in the Sea of Galilee (Lake Kinneret) ancient coastal settlements is influenced by the New Testament’s accounts of the miracles performed by Jesus. Thus, the study of the archaeological data originating from the excavation of the lake shores settlements offers an opportunity for tracing human reactions to past changes in lake level. Examples of major excavation come from; the Paleolithic settlement of Ohalo^5^, Bet Yerach^6^, El-Araj—the likely ancient Bethsaida-Julias of the early years of the 1st Millennium BCE^7^—as well as the Late Hellenistic-Early Roman harbor site of Magdala^8–10^. Another harbor site excavated is Kursi Beach (Fig. 1). In the early 1970s, archaeological surveys and excavations revealed harbor structures^11^. In 2015–2018, renewed excavations were undertaken and new data was published^12, 13^. Nevertheless, questions remain considering possible regional characteristics in harbor construction (at the scale of the lake) and the interconnection between the coastal sites.

The Sea of Galilee, in northern modern Israel (Fig. 1), is situated in a morphological depression along the Dead Sea Fault. It is the lowest freshwater lake on Earth (Lake surface at -210 m msl (mean sea level) in average). The area is characterized by a typical Mediterranean climate, with dry and hot summers and mild, wet winters. The lake is mainly fed by the Jordan River, entering it in the North, secondary wadis mainly active during winter and fresh and salt-water springs. The location of the lake along the Dead Sea Transform Fault renders it particularly sensitive to seismic hazards^14–19^ and major destructive earthquakes occurred in the area over time^20, 21^. This seismic activity is a major trigger of the stability of the slopes of the lake and the origin of numerous landslide^22, 23^. The area is also characterized by significant hydrological changes. While level changes of the Dead Sea have been intensively investigated^24–33^, there is a paucity of data relating to Sea of Galilee level fluctuations and their underlying causes—geologic, climatic or anthropogenic—during the Late-Holocene^34–36^.

**Figure 2:**
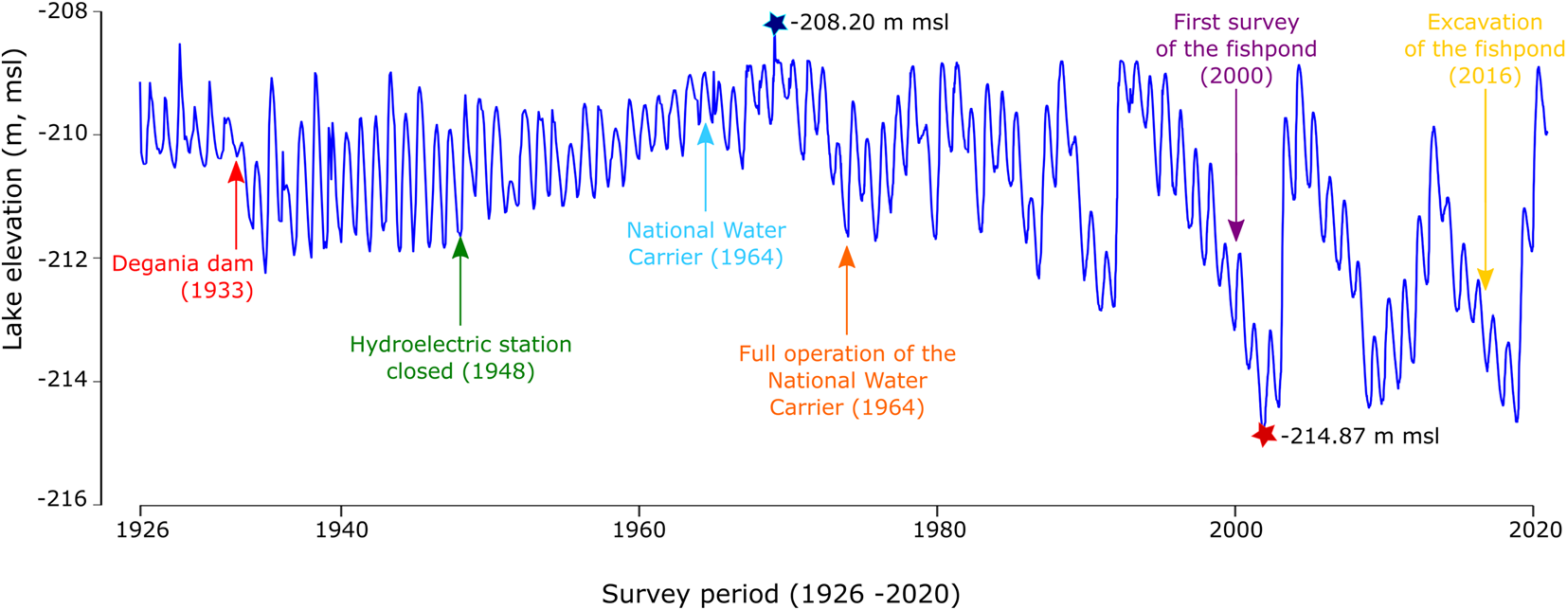
Mean monthly water levels of the Sea of Galilee between 1926 and 2020 (Data: Water Authority). Before the construction of the Degania dam (1933), the level of the sea fluctuated around -210 m msl. The magnitude of the fluctuations increased when the hydroelectric station was in operation. The use of the Sea of Galilee as freshwater reservoir further accentuated the fluctuations. The arrows represent the main interventions that have affected the natural variability of the lake level and the date of the excavation of the structure. The minimum water level has been recorded in November 2001 (red star; -214.87 m msl) and the maximum water level in February 1969 (blue star; -208.39 m msl).

Combination of both archeological and geological data have shown to be particularly useful in reconstructing changes in level of large inland lakes or seas such as Lake Turkana in Kenya^37–39^, the Aral Sea^40^, the Dead Sea e.g.^26,30,31^. For the Sea of Galilee, a lake level reconstruction has been proposed for the past 40,000 years^36^. The authors demonstrated that the Sea of Galilee level stabilized for long periods of time around -212 m msl. While most of the data covers pre-Holocene period, a maximum low-stand (<-212 m msl) has been evidenced in Early-Holocene (ca. 8000 years BP^36,37^). Major high-stand up to -200 m msl is also documented ca. 5000 years ago (Tel Bet Yerach^36,35^. A lake level of ca. -208/-211 m msl was also proposed according to the elevation of the early first century CE stadium of Tiberias^35–37^. However, the structure interpreted as the base of the stadium has recently been reassigned to be part of the Roman harbor^41^. Due to the presence of a tectonic fault passing through Tiberias’s archaeological site, one can question the use of the data as indicators of the lake’s level changes. Over the past century, human impact is the main cause of lake level fluctuations^42^ (Fig. 2).

**Figure 3:**
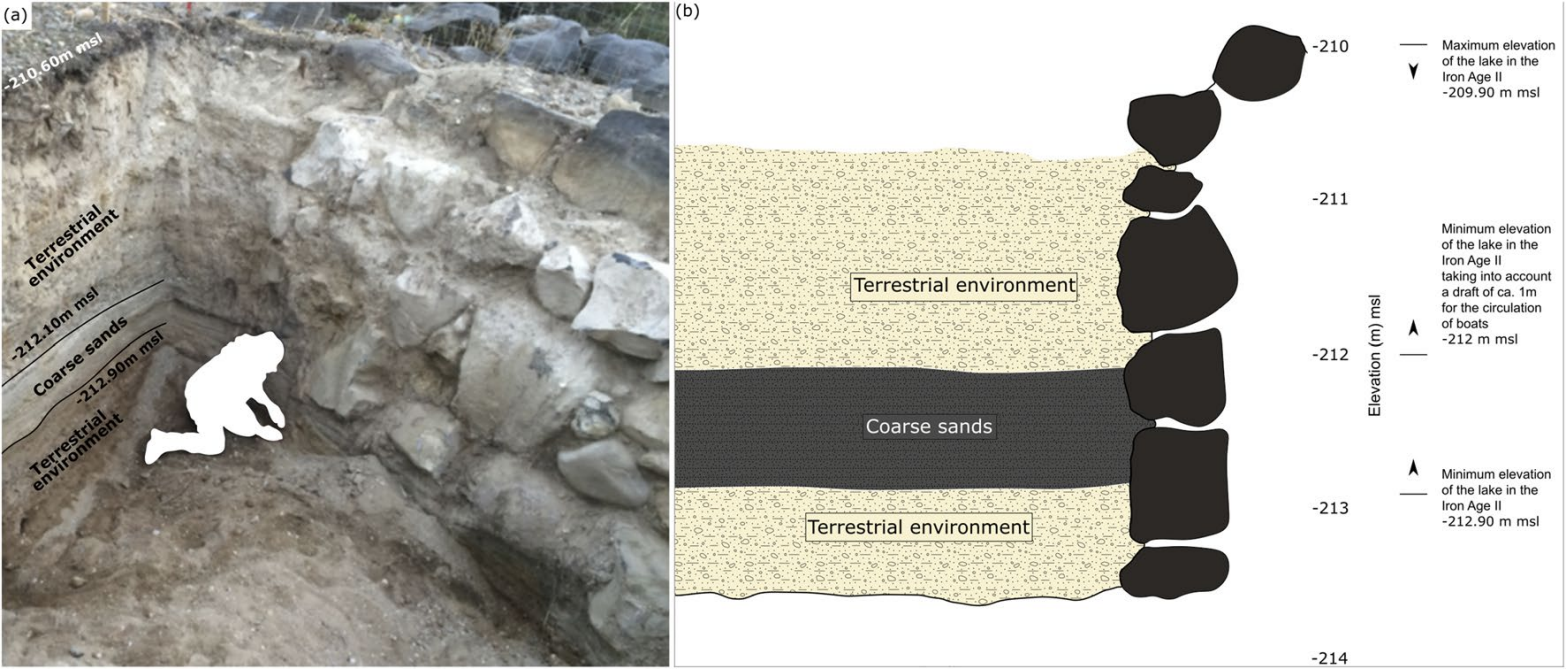
The breakwater. (**a**) Photo of the trench in the inner face of the breakwater and (**b**) drawing of the sedimentary units with estimated ancient lake level elevation. The three environmental units have been differentiated by their sedimentary texture and color (details of the sedimentary units are given in Supplementary text).

**Figure 4:**
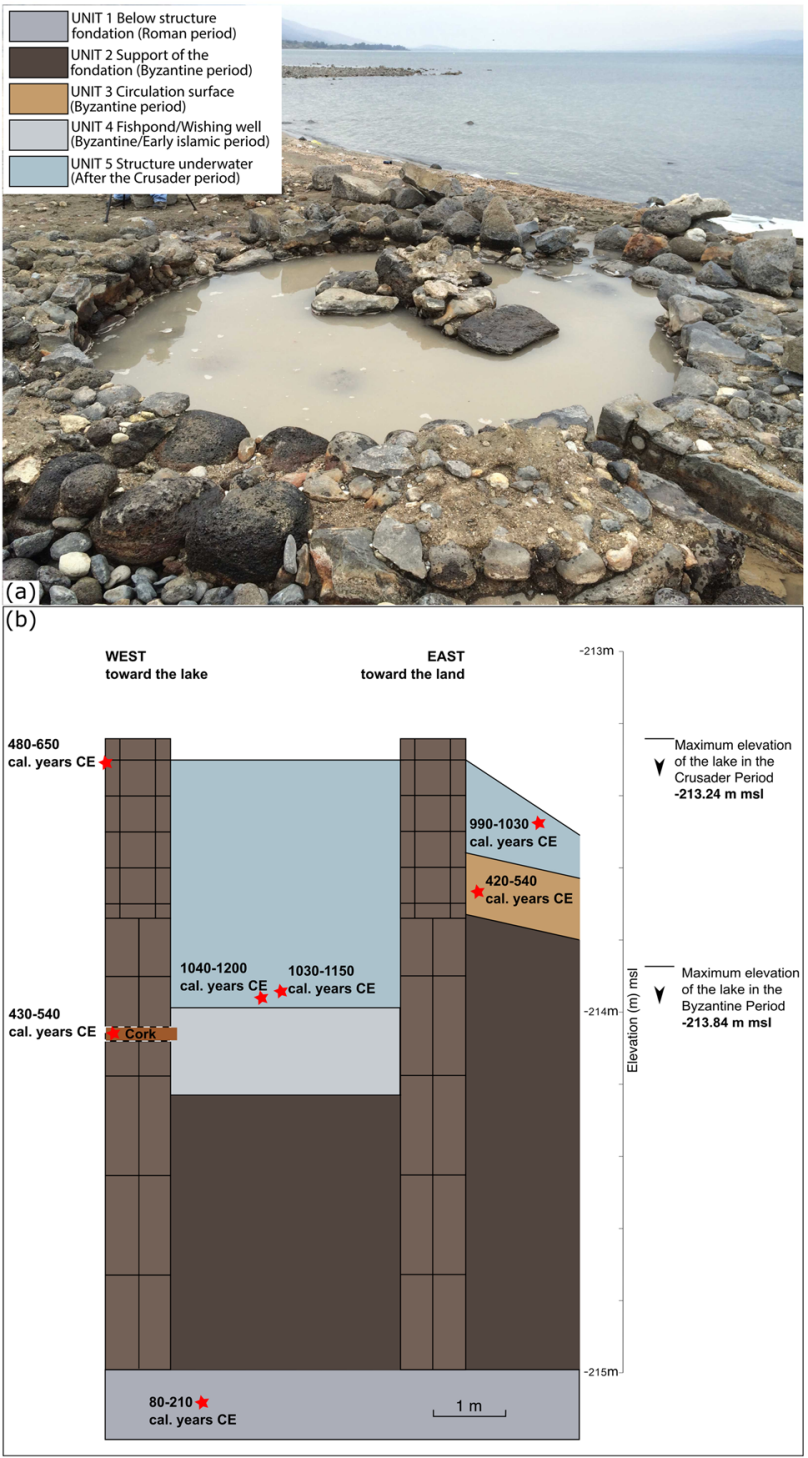
The fishpond/wishing well (Area C). (**a**) Structure viewed from the North with the lake in the back (Photo: Michal Artzy) and (**b**) Lake-level index points obtained from the study of the fishpond. The red stars represent the location of the samples dated by radiocarbon. The index points are compared and contrasted with data originating from other sites in Fig. 5.

**Figure 5:**
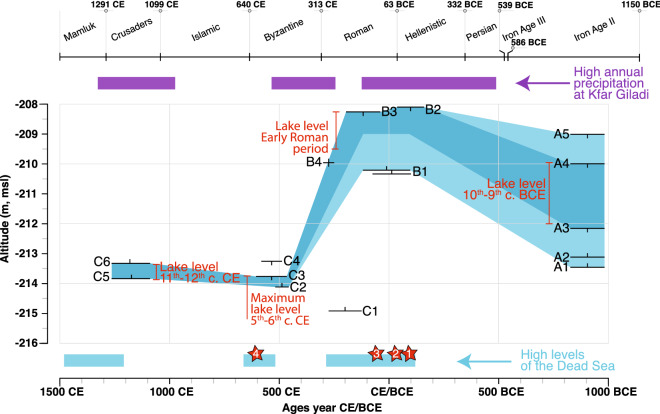
Sea of Galilee water level index points and lake level envelope. The red vertical bar indicates the position of the water (A1–A5 present the data from the breakwater/harbor basin at Kursi (A1 = base of the breackwater ; A2 = base of the harbor unit; A3 = top of the harbor unit; A4 = top of the breakwater at the time of excavation; A5 = estimated elevation of the breackwater in the Iron Age II); B1-B3 present the data from the harbor at Magdala^44^  (B1 = bottom of the harbor basin; B2 = Late-Hellenistic mooring stone; B3 = Early Roman mooring stone; B4 = Late Roman landing zone); C1-C6 present the data from the fishpond at Kursi (C1 = locus 28011; C2 = cork; C3 = circulation surface; C4 and C6 = structure elevation ; C5 = infilling dated from the crusader period). The indicators show high lake level during the Iron Age II and the Late Hellenistic/Early Roman period (between -208 and -210 m msl) and low lake levels during the Byzantine, Early Islamic and Crusader periods (<-213 m msl). Reconstructed high precipitation at Kfar Giladi (>600 mm/year) from Morin et al.^33^  and high Dead Sea levels (>-402 m msl, corresponding to the sill elevation) from Kushnir and Stein^32^. Red stars represent specific high-water level of the Dead Sea reported from ancient authors or maps by Binder et al.^31^

Kursi Beach archaeological site (Site 12, Horbat Kursi, Harbour^43^) is one of the numerous coastal sites identified on the shore of the lake^11, 16^. It is located in the Kursi Beach Nature Reserve, on the fringes of the western Golan Heights along the eastern shore of the Sea of Galilee (Fig. 1). Archaeological investigations have allowed the excavation of coastal structures suggesting fluctuations in past lake levels^12, 13^ (Fig. 1). A breakwater (Figs.1 and 3) and a fishpond (Figs. 1 and 4), as well as key *loci* uncovered during the excavations are used to provide relative lake level index points (details of the different *loci* are given in Supplementary Text). Using data originating from the Kursi Beach excavations and data gathered from archaeological structures on the Lake’s shore, our aim is to improve the resolution of the Late-Holocene lake level history^31–37^. Our data allow fill in a gap in antiquity, a period of high interest for the archaeological community by providing original and precise lake level index point (Fig. 5).

## Results and discussion

Lake shores can be affected by quick and recurrent (seasonal) fluctuations of water level due to climatic (e.g. variations in distribution and intensity of precipitations), geological (e.g. tectonic activity, slope movements) or anthropogenic factors acting at local or regional scale. The combination of historical, archaeological and geomorphological indicators is particularly well adapted to study these changes in the case of the Sea of Galilee. Our new data from Kursi Beach allow to highlight lake level elevation from the Iron Age II to the Mediaeval period.

### Intermediate lake levels in the Iron Age II period (10th-9th centuries BCE)

Elevation of the breakwater has been used to reconstruct lake level position in the Iron Age II period (Figs. 3 and 5). To understand the construction of the breakwater and to know its depth, a trench, perpendicular to it, was open up to a depth of 3.5 m (top of the trench at -210.60 m msl; Fig. 3). It was oriented in a general southeast-northwest direction in accordance with the angle formed by the inner face of the breakwater (Fig. 1c). The inner face of the breakwater, which was exposed in the trench, reveals that it was built of large basalt stones. On its outer face, facing the lake, the stones were arranged diagonally toward the West. On the inner face (average height of 2.5 m), the stones were carefully placed vertically (Fig. 3). The new excavation found that the breakwater was built to a maximum elevation of -210 m msl. It was assumed that the breakwater should have been at least 1 m higher during the period of its usage. However, even at -209 m msl, the elevation of the breakwater did not allow its optimal utilization during the Late Hellenistic/Early Roman period when the level of the lake was around -208/-209 m msl (Fig. 5). Since the eastern shore of the lake is affected by >1.5 m high waves^45^, the structure must have been much higher than that of the lake level. Our data demonstrate that the dating of the breakwater to the Hellenistic period or later as suggested by Raban^11^ is problematic. This dating only relies on similarities with other Mediterranean harbor-works dated from the same period. While we still lack data regarding the chronology of the infilling of the basin, the absolute absence of remains dated from the Hellenistic period found during the 2015–2018 excavations point toward an absence of coastal activities in the area indicating a pre-Hellenistic infilling of the inner harbor^12, 13^. In contrast, we can reasonably assume that the breakwater was in use during the Iron Age II period or earlier, concurrent with the settlement of Tel Kursi dated to the 10th–9th centuries BCE by ceramic finding and radiocarbon (Table 1; Figure S1). According to our calculation, water level was between -209.90 and -212 m msl during the Iron Age II period, allowing the inner harbor basin to function. This relative high lake level also corresponds to a period of low, but rising Dead Sea level^30, 32, 34^.

**Table 1:** AMS-14C data expressed in calibrated years BP and BCE/CE at the 95% confidence level (2σ). We used Calib 8.2^46,47^ and the IntCal20 curve^48^. Sample RT 4447 from Galili et al.^45^.

Sample	Lab number	Material	Elevation (m mean sea level)	Age 14C	2 sigma cal. BP min–max	2 sigma cal. BCE/CE min–max
L.44206	ULA-9104	Charcoal		2760 ± 15	2780–2920	970–830 BCE
L.44208(2)	ULA-9103	Charcoal		2740 ± 15	2780–2860	920–830 BC
L.28011	ULA-9105	Plant remains	-214.98	1900 ± 15	1740–1870	80–210 CE
L.28105	ULA-9102	Wood	W -213.53/-213.63 E -213.36/-213.46	1015 ± 15	920–960	990–1030 CE
L.28107	ULA-9134	Grape seed	W -213.73/-213.84 E -213.56/-213.67	1595 ± 15	1410–1530	420–540 CE
L.28202	ULA-9127	Plant remains	-213.95/-213.98	920 ± 15	750–910	1040–1200 CE
L.28203	ULA-9106	Plant remains	-213.95/-213.99	960 ± 15	800–920	1030–1150 CE
Cork L.28205	ULA-9129	Wood	-213.97/-214.08	1590 ± 15	1410–1520	430–540 CE
RT 4447		Remains of carbonized wood and grain seeds		1480 ± 40	1300–1470	480–650 CE

### High lake levels in the Late Hellenistic/Early Roman period

At Kursi, shallow lakeshore type sediments dated to the Late Hellenistic/Early Roman period found at -214.98 m msl (below the fishpond foundations; Figs. 4 and 5; Figs. S2 and S3) demonstrate that the level of the lake was higher at that period. At Magdala (on the western shore of the lake), data originating from the excavation of the harbor basin further indicate that the level of the lake was between -210.30 m msl (bottom of the basin) and -208.30 m msl (elevation of the mooring stone) for that period^44^. This high level corresponds to a period of increased precipitation in the southern Levant in the interval 130–40 BCE and coincide with high level of the Dead Sea around -394 m msl in the 1st century CE^31–33^. A landing surface excavated at Magdala, suggests that the water level dropped to ca. -210 m msl during the second half of the 3rd century CE^44^. Excavations at Galei Kinneret (ancient Tiberias), south of Magdala, have exposed buildings dated from the Roman period^35^, likely part of the Roman harbor^41^. Linking lake level with the elevation of the structures, it was proposed that the harbor was built in the 1st century and used until the 3rd century CE when a drop in the water level led to the abandonment—and displacement—of the harbor area. However, due to the presence of tectonic faults passing through the site^35^, the structures cannot be used to provide direct lake level index point.

### Low lake levels from the Byzantine to the Crusader period

The fishpond was first identified during a survey in the autumn 2000 following a drop in lake level to -213.80 m msl^45^. After a rise in lake’s water level, the structure remained underwater until 2016, when the level of the water reached the elevation of -213.70 m msl allowing for its excavation (Fig. 2). The top of the structure is at an elevation of -213.24 m msl (Fig. 4, Figures S2 and S3), although it was likely higher since many loose stones belonging to it were found in the area. The structure is round, ca. 5 m diameter (Figs. 4 and S2). The width of the wall is ca. 90 cm. A third of the structure was excavated and it was found out that the foundation stones—un-hewn basalt stones—were placed in such a way that each layer protrudes ca. 2 cm (Fig. S2). It was built without bonding material. The foundation stones were placed against a wet surface composed of dark organic mud. Above the foundation walls, were two layers of small and medium stones held together by plaster made of small stones, organic material and lime (Fig. S2). In the lower level, three openings were noted: North, West and East. These openings are conical and the thin opening is facing inwards. In the western opening’, which is slightly lower than the others, a ‘cork’ like log, in the shape of the opening, was found (Figure S4). The construction method of the 5th–6th centuries CE fishpond discovered at Kursi shows that it was constructed when the water level was lower or similar to that of late summer/early autumn 2016 (-213.70 m msl). As the fishpond was likely partially filled with the water originating from a local spring, the level could have even been lower. The circulation surface discovered in the square excavated outside the structure (-213.56/-213.84 m msl) is contemporaneous to that of the structure and the wooden cork found at -214.08 m msl. Thus, the lower elevation of the floor—at -213.84 m msl—can be considered as the maximum mean elevation of the lake at the time of the construction. At the same period (5th–early 6th century CE), the Dead Sea knew a pluri-metric drop of its level before an important rise at the end of the 6th century CE^31,32,25^. At Kursi, our research demonstrates an important drop in lake level between the Roman and the Byzantine period as attested by the fishpond elevation. At Magdala, this drop is confirmed by the likely presence of coastal structures dated to the Late Byzantine/Early Islamic period at three meters lower than that of the Roman harbor structures^44,27^. The sharp, but relatively limited in time, increase attested in the level of the Dead Sea around 550 ± 50 CE is not visible in our record for the Sea of Galilee. Indeed, large number of coins, mainly dated to the Early Islamic period, were found inside the structure. This attests to the fact that the water of the lake was likely still lower than -213.70 m msl allowing pilgrims visiting the nearby monastery to use the fishpond as a wishing well. On the western shore of the lake, archaeological structures (at Galei Kinneret, the ancient Tiberias) buried under lake sediments indicate a rise in lake level during the late-8th century CE. This rising level has been attributed to tectonic activities and the consequence of the 789 CE earthquake affecting the western shore of the lake^35^. Data from Kursi indicate that the lake may have remained at a low elevation until the Crusader period in agreement with a low-stand of the Dead Sea between the 8th and the 11th centuries CE^26,32,25^. Shallow lakeshore type sediments found inside the structures—covering the levels containing Early Islamic coins—are dated to the 11th–12th centuries CE (Fig. 5; Supplementary text). This rising level puts an end to the utilization of the wishing well. Thus, no coins dated after the Early Islamic period have been found in the upper layers of the structure infill. However, the coexistence of aquatic and air-breathing mollusks further indicate that the structure may not have remained permanently underwater.

### How people dealt with lake level changes?

Many coastal structures have been noted along the shores of the Sea of Galilee, yet not all have been clearly dated. It is assumed that a large number were constructed during the Hellenistic/Roman/Byzantine periods^49, 50^. These structures are built of local undressed boulders, reflecting the water elevation at the time of construction^50^. They are generally modest and required minimal human and economic investment. The discovery of the structures at different elevations—most of them between -208 and -214 m msl—may imply that they could be easily abandoned or modified to accommodate lake level variations^51^.

At Kursi, coastal structures dated from different archaeological periods found at different elevations on the shore of the lake confirm that the ancient inhabitants built the structures to accommodate the changing level of the lake. Our data demonstrates that lake level was likely between -210 and -212 m msl during the 11th–12th centuries BCE. A low lake level (<-213.80 m msl) has been identified from the Byzantine (5th–6th centuries CE) to the Crusader period (11th–12th centuries CE). The dating of the breakwater of Kursi to the Iron Age II, or even earlier, makes it the oldest artificial coastal structure dated in the Sea of Galilee. It is not surprising as the Bronze and Iron Age saw the flourishing of important settlements (e.g. Bet Yerah, Tel Hadar and Bethsaida). This structure is the largest discovered along the shore of the lake. The breakwater of Kursi is remarkable because of its dimensions and because it is of good structural quality in comparison with light coastal structures identified along the shore^11, 51^. It measures more than 140 m in length and has a high of 2.5 m in its southern extremity. Test pits opened in its northern part suggest that its height could have reached 4.5 m. According to our estimations, the maximum water column of the southern shore of the inner harbor was ca. 2 m allowing the consideration of seasonal, and maybe pluri-annual, fluctuations in lake level. However, the depth of the water may have been greater further north, toward the center of the basin. We do not know when the usage of the anchorage of Kursi ended. Regarding the lack of data to date the infilling of the harbor basin, its abandonment could have been linked to change in the level of the lake or to the infilling of the basin as for many other harbors in the Mediterranean^53,52^. Changes in geopolitical vicissitudes effecting trade networks should also be considered. At Magdala, geoarchaeological investigation of the harbor area have shown that the reorganization of the harbor infrastructures has likely been undertaken to accommodate coastal progradation and small-scale changes in lake level during the Hellenistic and Roman periods^44^. The apparent stability of the lake level from the Late Hellenistic to the Early Roman period and the associated coastal progradation lead to the construction of two successive quays parallel to each other^9, 44^. The likely low elevation of the lake from the Byzantine to the Crusader period indicated by the fishpond/wishing well elevation further questions the pluri-annual variations in the elevation of the lake. This apparent stability of the water level contrasts with fluctuations of the Dead Sea level during that period, specifically regarding the high level recorded in the 6th century CE^25,30,31^.

## Conclusion

Water stress poses serious threats to human lives and livelihoods. The current situation regarding access to freshwater resources is poised to worsen unless countries act. Today, as in ancient times, population growth, socioeconomic development and urbanization are increasing water demand, while climate change can make precipitations and water availability more variable. Reconstructing past water availability and uses through the reconstruction of water quantity in continental reservoirs is challenging. Our research poses the basis for future investigations regarding lake level changes of the Sea of Galilee. We highlight that lake level fluctuations must have been key factors taken into consideration by local populations. The important number of sites on the shore of the Sea of Galilee and the ongoing discovery and excavation of sites would provide new data. This is, for example, the case of the multi period (Roman, Byzantine and Crusader) site of El-Araj/Bethsaida, buried below several meters of sediments in the delta of the Jordan river, in the northern side of the lake^7^. In relatively flat areas as for the northern Jordan delta, even small fluctuations in lake level may have led to important displacement of the shoreline and of the harbor activities.

The present study contributes to a better understanding of Sea of Galilee’s water level changes from the 1st Millennium BCE to the early-2nd Millennium CE, a period of high interest for the archaeological community. Our data from the Kursi Beach excavation demonstrates that lake level was likely between -210 and -212 m msl during the 10th-9th centuries BCE. A low lake level (<-213.80 m msl) has been identified from the Byzantine (5th–6th centuries CE) to the Crusader period (11th–12th centuries CE). Data from Magdala demonstrate a high-stand of lake level around -208.30 m msl during the Late Hellenistic/Early Roman period^44^. The association of archaeological and bioarcheological indicators is particularly useful to infer lake level position at a certain period and its changes through time.

Multiplication of geoarchaeological studies and precise dating of the coastal structures will produce more lake level indicators and will increase the spatial and temporal resolution of the record. Thus, the availability of a more detailed curve of lake level would provide data to be compared and contrasted with local or regional climatic reconstructions to decipher the impact of climate and the possible human influence on the changes in lake levels. Archaeological excavation of well dated coastal structures would further improve our understanding of population adaptation to seasonal and pluriannual changes in water level. In the current state of our understanding, direct human intervention in water availability in lake reservoirs in the Jordan Valley has not been evidenced in ancient and early mediaeval times. The integration of seasonal and pluri-annual variations in the lake level within coastal urbanization strategies is at the crux of discussions concerning the adaptation of the populations to the changes.

## Material and methods

### Archaeological excavations and dating of the structures

Archaeological excavations at Kursi Beach have been undertaken by M. Artzy and H. Cohen between 2015 and 2018 under the auspices of the Hatter Laboratory, RIMS, University of Haifa. In this study, we focus on areas A and C in the excavation (Fig. 1), where coastal structures have been identified. Elevation of the structures has been measured with a Differential Global Positioning System (DGPS). The dating of the structures is based on the archaeological evidences (coins, ceramics, and construction techniques). In addition, eight Accelerator Mass Spectrometry (AMS) radiocarbon dating have been performed. The preparation of the samples, which includes chemical pre-treatment, fractionation of their carbon components followed by oxidation and reduction to graphite was completed at the Radiochronology Laboratory of the *Centre d’Études Nordiques* (University of Laval, Canada). AMS measurements were performed at the Keck Carbon Cycle AMS Facility (University of California-Irvine, USA; Table 1). Samples submitted were charcoals, grape seeds, plant remains or wood. Dates were calibrated using Calib 8.2^47,46^ and IntCal20 curve^48^. The dating of Galili et al.^45^, indicates that the structure was built under the Byzantine Rule (313–636). Calibration of the radiocarbon date (1480 ± 40 BP) obtained from remains of carbonized wood and grain seeds extracted from the mortar gives an age of 480–650 cal. years CE.

### Sedimentological and faunal analysis

Sediments collected in the structures have been analyzed to establish their texture and their faunal content. The general sediment texture includes gravel (>2 mm), sand (63–2 mm) and silty-clay fractions (<63 μm). It was determined by the wet sieving of 100–150 g of dry sediments. Mollusks have been identified in the gravel fraction. Species were determined using existing literature^54^. Ostracods were extracted from the fraction >125 μm and identified to species when possible^56,55^. The general description of the loci is provided in Supplementary text.

### Reconstruction of lake level elevation

We use archaeological structures to provide indicators of relative lake level. The index points are based on the elevation of the structure according to present global mean sea level and the estimation of the lake level needed for their usage. Although it is challenging to define the exact elevation of the lake level at specific periods, we provide general envelope of lake level elevation or propose a relative elevation of the lake level (higher or lower) according to the structure and the sediment collected during their excavation (grain size and faunal content).

## Supplementary Information

Supplementary Information.

## References

[1] Bar-Yosef O, Fitzhugh B, Habu J (2002). Natufian. Beyond Foraging and Collecting.

[2] Byrd BF, Kuijt I (2002). Households in transition. Life in Neolithic Farming Communities.

[3] Bar-Yosef O, Belfer-Cohen A, Gebauer AG, Price TD (1992). From foraging to farming in the Mediterranean Levant. Transition to Agriculture in Prehistory.

[4] Mithen S, Black E (2011). Water, life and civilisation: Climate, environment and society in the Jordan Valley.

[5] Nadel D (2002). Ohalo II-a 23,000 Year-Old Fisher-Hundter-Gatherers’ Camp on the Shores of the Sea of Galilee.

[6] Greenberg R, Paz S, Wengrow D, Iserlis M (2012). Tel bet yerah: Hub of the early bronze age levant. Near Eastern Archaeol..

[7] Notley RS, Aviʿam M (2020). Searching for Bethsaida: The case for El-Araj. Biblic. Archaeol. Rev..

[8] Avshalom-Gorni, D. & Najar, A. Migdal, preliminary account. *Hadashot Arkheologiyot* **125**. http://www.hadashot-esi.org.il/report_detail_eng.aspx?id=2304&mag_id=120 (2013).

[9] De Luca S, Lena A, Ladstätter S, Pirson F, Schmidts T (2014). The Harbor of the City of Magdala/Tarichæe on the shores of the Sea of Galilee, from the Hellenistic to the Byzantine times. New Discoveries and Preliminary Results. Harbors and Harbor Cities in the Eastern Mediterranean from Antiquity to Byzantium. Recent Discoveries & New Approaches.

[10] Bauckham R, De Luca S (2015). Magdala as we now know it. Early Christ..

[11] Raban A (1988). The boat from Migdal Nunia and the anchorages of the Sea of Galilee from the time of Jesus. Int. J. Naut. Archaeol..

[12] Cohen, H. & Artzy, M. Kursi Beach–2015: preliminary report. *Hadashot Arkheologiyot* **129**. https://www.hadashot-esi.org.il/report_detail_eng.aspx?id=25247&mag_id=125 (2017).

[13] Artzy M, Cohen H, Misgav H (2019). Hafirot Hof Kursi: The Synagogue and the Inscription found there. Qadmoniyot.

[14] Sadeh M (2012). Crustal deformation along the dead sea transform and the carmel fault inferred from 12 years of GPS measurements. J. Geophys. Res. Solid Earth..

[15] Singer A, Gal M, Banin A (1972). Clay minerals in recent sediments of Lake Kinneret (Tiberias), Israel. Sediment. Geol..

[16] Nun, M. Sea of Galilee: Newly Discovered Harbours from New Testament Days, Kinnereth Sailing, Kibbutz Ein Gev (1989).

[17] Amiran DH, Arieh E, Turcotte T (1994). Earthquakes in Israel and adjacent areas: macroseismic observations since 100 BCE. Israel Expl. J..

[18] Ferrario MF (2020). The mid-eighth century CE surface faulting along the dead sea fault at tiberias (Sea of Galilee, Israel). Tectonics.

[19] Gasperini L (2020). Neotectonics of the Sea of Galilee (northeast Israel): implication for geodynamics and seismicity along the Dead Sea fault system. Sci. Rep..

[20] Russell KW (1985). The earthquake chronology of Palestine and northwest Arabia from the 2nd through the mid-8th century AD. Bull. Am. Sch. Orient. Res..

[21] Marco S, Hartal M, Hazan N, Lev L, Stein M (2003). Archaeology, history, and geology of the AD 749 earthquake, Dead Sea transform. Geology.

[22] Katz O (2009). Quaternary earthquakes and landslides in the Sea of Galilee area, the Dead Sea Transform: Paleoseismic analysis and implication to the current hazard. Israel J. Earth Sci..

[23] Yagoda-Biran G, Hatzor YH, Amit R, Katz O (2010). Constraining regional paleo peak ground acceleration from back analysis of prehistoric landslides: example from Sea of Galilee, Dead Sea transform. Tectonophysics.

[24] Bartov Y, Stein M, Enzel Y, Agnon A, Reches ZE (2002). Lake levels and sequence stratigraphy of Lake Lisan, the late Pleistocene precursor of the Dead Sea. Quatern. Res..

[25] Frumkin A, Elitzur Y (2002). Historic dead sea level fluctuations calibrated with geological and archaeological evidence. Quatern. Res..

[26] Bookman R, Enzel Y, Agnon A, Stein M (2004). Late Holocene lake levels of the Dead Sea. Geol. Soc. Am. Bull..

[27] Migowski C, Stein M, Prasad S, Negendank JF, Agnon A (2006). Holocene climate variability and cultural evolution in the Near East from the Dead Sea sedimentary record. Quatern. Res..

[28] Neugebauer I (2014). Lithology of the long sediment record recovered by the ICDP Dead Sea Deep Drilling Project (DSDDP). Quatern. Sci. Rev..

[29] Kagan EJ, Langgut D, Boaretto E, Neumann FH, Stein M (2015). Dead Sea levels during the Bronze and Iron ages. Radiocarbon.

[30] Binder SE, Lazar M, Nantet E (2019). Measurements and shape of the Dead Sea in the hellenistic and roman periods: Confronting greek and latin sources with modern physiographical data. Israel Expl. J..

[31] Kushnir Y, Stein M (2019). Medieval climate in the eastern mediterranean: Instability and evidence of solar forcing. Atmosphere.

[32] Morin E, Ryb T, Gavrieli I, Enzel Y (2019). Mean, variance, and trends of Levant precipitation over the past 4500 years from reconstructed Dead Sea levels and stochastic modeling. Quatern. Res..

[33] Weber N, Lazar B, Gavrieli I, Yechieli Y, Stein M (2021). Gypsum deltas at the holocene dead sea linked to grand solar minima. Geophys. Res. Lett..

[34] Hazan N, Stein M, Marco S (2004). Lake Kinneret levels and active faulting in the Tiberias area. Israel J. Earth Sci..

[35] Hazan N, Stein M, Agnon A, Neev D (2005). The late quaternary limnological history of Lake Kinneret (Sea of Galilee), Israel. Quat. Res..

[36] Lev L, Stein M, Ito E, Fruchter N, Ben-Avraham Z, Almogi-Labin A (2019). Sedimentary, geochemical and hydrological history of Lake Kinneret during the past 28,000 years. Quatern. Sci. Rev..

[37] Owen RB, Barthelme JW, Renaut RW, Vincens A (1982). Palaeolimnology and archaeology of Holocene deposits north-east of Lake Turkana, Kenya. Nature.

[38] Bloszies C, Forman SL, Wright DK (2015). Water level history for Lake Turkana, Kenya in the past 15,000 years and a variable transition from the African Humid Period to Holocene aridity. Global Planet. Change.

[39] Wright DK, Forman SL, Kiura P, Bloszies C, Beyin A (2015). Lakeside view: Sociocultural responses to changing water levels of Lake Turkana, Kenya. Afr. Archaeol. Rev..

[40] Boroffka N, Oberhänsli H, Sorrel P, Demory F, Reinhardt C, Wünnemann B, Alimov K, Baratov S, Rakhimov K, Saparov N, Shirinov T, Krivonogov SK, Röhl U (2006). Archaeology and climate: Settlement and lake-level changes at the Aral Sea. Geoarchaeology.

[41] Bonnie R (2017). From stadium to harbor: Reinterpreting the curved ashlar structure in Roman Tiberias. Bull. Am. Sch. Orient. Res..

[42] Hambright KD, Eckert W, Leavitt PR, Schelske CL (2004). Effects of historical lake level and land use on sediment and phosphorus accumulation rates in Lake Kinneret. Environ. Sci. Technol..

[43] Hartal, M. & Ben Efraim, Y. Map of Ein Gev (40). Archaeological Survey of Israel. http://www.antiquities.org.il/survey/new/default_en.aspx?surveynum=38 (2012).

[44] Sarti G (2013). Magdala harbour sedimentation (Sea of Galilee, Israel), from natural to anthropogenic control. Quatern. Int..

[45] Galili, E., Rosen, B., Boaretto, E. & Tzatzkin, S. Kursi Beach. Hadashot Arkheologiyot 119. https://www.hadashot-esi.org.il/report_detail_eng.aspx?id=25247&mag_id=125 (2007)

[46] Stuiver M, Reimer PJ (1993). Extended 14C database and revised CALIB radiocarbon calibration program. Radiocarbon.

[47] Giaime M, Marriner N, Morhange C (2019). Evolution of ancient harbours in deltaic contexts: A geoarchaeological typology. Earth Sci. Rev..

[48] Stuiver, M., Reimer, P. J. & Reimer, R. W. CALIB 8.2 [WWW program] at http://calib.org. Accessed 17 Aug 2020 (2020).

[49] De Luca S (2010). La città ellenistica-romana di Magdala/Taricheae. Gli scavi del Magdal Project 2007 e 2008: relazione preliminare e prospettive di indagine. Liber Annuus.

[50] Nun M (1977). Sea of Kinneret, a Monograph.

[51] Galili E, Sharvit J (2002). The sea of galilee. Coastal and underwater surveys. Hadashot Arkheologiyot.

[52] Galili E, Arenson S (2014). Management of the underwater and coastal archaeological heritage in Israel’s seas (I). Riparia.

[53] Morhange C (2015). Dynamiques géomorphologiques et typologie géoarchéologique des ports antiques en contextes lagunaires. Quaternaire.

[54] Reimer PJ (2020). The IntCal20 Northern Hemisphere radiocarbon age calibration curve (0–55 cal kBP). Radiocarbon.

[55] Tchernov E (1975). The molluscs of the Sea of Galilee. Malacologia.

[56] Lerner-Seggev R (1968). The fauna of ostracoda in lake Tiberias. Isr. J. Zool..

[57] Meish C (2000). Freshwater Ostracoda of western and central Europe.

