# Supplementary material for: Using archaeological data for the understanding of Late-Holocene Sea of Galilee’s level fluctuations
Source: Sci Rep. 2022 Jun 13;12:9775. doi: 10.1038/s41598-022-09768-8 (PMC9192592; doi:10.1038/s41598-022-09768-8)
Supplement: Supplementary file 1 — Supplementary Information. [file 41598_2022_9768_MOESM1_ESM.pdf]

Supplementary Text

The breakwater

Sediments collected in the trench have been analyzed in the laboratory. No fauna was found. However, three different units were distinguished based on the texture and the color of the sediments. Organic remains were quasi absent from the sediments. Only one charcoal piece was found and dated.

Between -213.40 and -212.90 m msl, samples are mainly composed of sands (50%) with 29% of fine sediments and 21% of gravels. The sediments appear to be from terrestrial origin due to their color and the presence of tiny roots. The charcoal, collected between 310 and 315 cm depth has been dated to 3855 ± 15 BP (2450 – 2210 cal. years BCE).

Between -212.90 and -212.10 m msl, the general texture changes. The sediments are composed of sands (78%) with 17% of gravels and only 5% of fine sediments. These medium to coarse grey sands reflect the presence of an underwater environment.

Between -212.10 and -210.20 m msl, the general texture changes again. The sediments are still mainly composed of sands (64%), the proportion of gravels reached 28% and the proportion of fine sediments increases to 8%. Presence of tiny roots and the yellowish color of the sediments reflects their terrestrial origin.

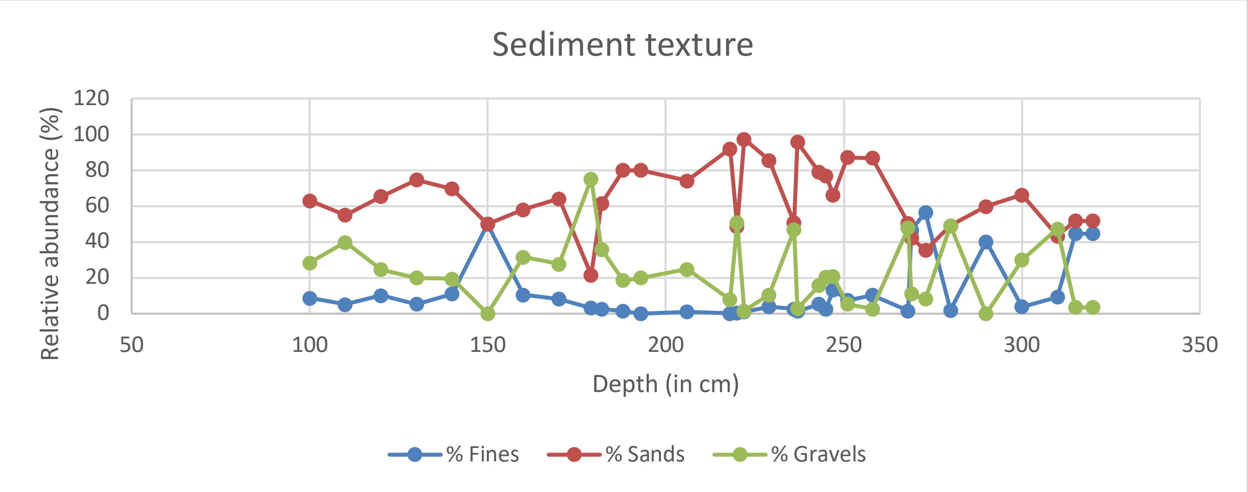

Figure 1: Sediment texture of the samples collected in the trench behind the breakwater

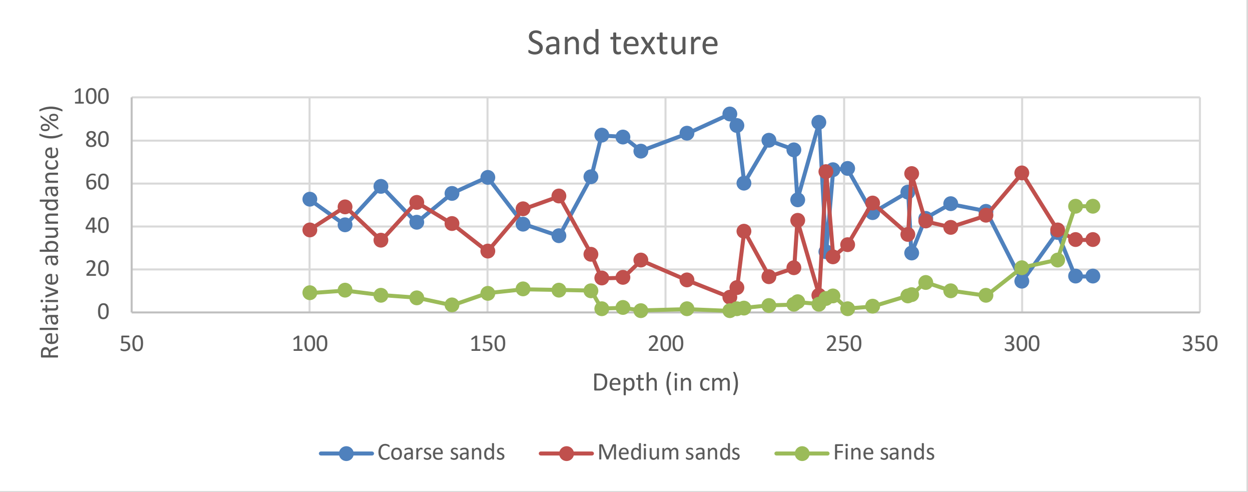

Figure 1: Sand texture of the samples collected in the trench behind the breakwater

The fishpond/wishing-well

Locus 28011 is located below the structure's foundation (-214.98/-215.03 m msl). It is composed of sands (61%), gravels (29%) and silty-clay (10%). No mollusks have been found. The ostracod assemblage is the most diverse but density is low with 113 valves for 10 grams of sediments. It is dominated by *Potamocypris sp.* (24%), *Eucypris virens* (18%), *Ilyocypris* spp. (18%) and *Pseudocandona* sp. (11%). This locus is dated to the Roman period (1900 ± 15 BP; 80 – 210 CE). Fauna is characteristic of a lake shore. *Eucypris virens* is, however, reported from temporary water bodies, spring, streams and fishponds (Meish, 2000).
This locus is interpreted as a lake shore deposit in the Roman period. The presence of E. virens may reflects the existence of the wadi outlet or a spring nearby. This locus is used as a lake limiting point (Fig. 3).

Locus 28010 is a 75cm thick unit, located between -214.23 and -214.98 m msl. It is mainly composed of silts and clays (83%) with 15% of sands and 2% of gravels. Mollusks are extremely rare and only represented by two individuals of *Melanopsis praemorsum* and one individual of *Theodoxus* sp. The ostracod density is high with 466 valves for 10 grams of sediments. *Pseudocandona* sp. (28%), *Ilyocypris* sp. (25%), *Potamocypris* sp. (20%) and *Cyprideis torosa* (15%) dominate the assemblage. *Pseudocandona* sp. has a wide distribution and is frequently found in lakes and pounds on fine sediments. As well as *Ilyocypris* sp. and *C. torosa*, it is found with relatively high frequencies in both standing and running waters (Mischke et al., 2012).
This locus is located at the bottom of the structure into which the foundations were placed. During excavation, this layer has been found to be waterproof. Ostracod assemblage and sediment texture of the unit differs from the species commonly encountered on the shore of the lake. One possibility is that the sediment was intentionally deposited in this area for the construction of the structure.

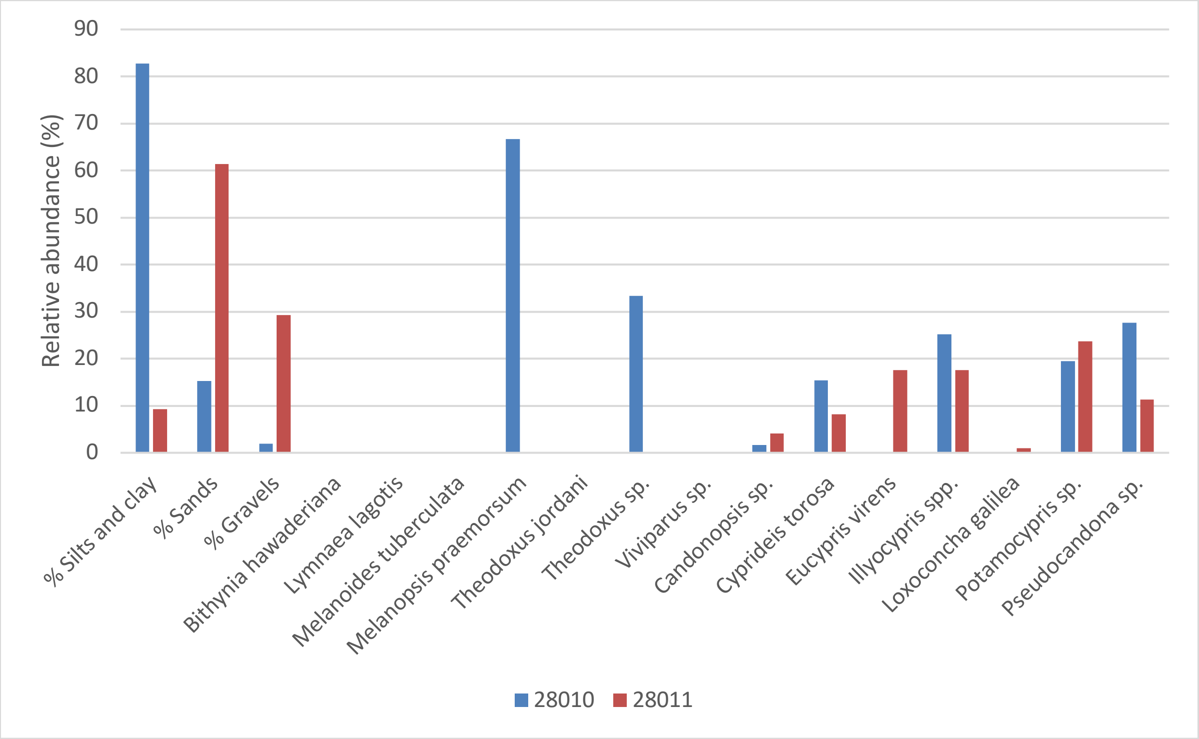

Figure 3: Sediment texture and fauna for locus 28010 and 28011

Loci 28205, 28203 and 28202 (Fig. S2) have a similar sedimentary texture. They are mainly composed of sands (59-68%) with gravels (15-34%) and contain 5-17% of silts and clay. Mollusks assemblage of loci 28205 and 28202 is dominated by *T. jordani* (72-50%), followed by *M. praemorsum* (18-23%). Locus 28203 is dominated by *Viviparus* sp. (40%), T. jordani (30%), *M. praemorsum and L. lagotis (10%). Viviparus* and L. *lagotis* are air-breathing freshwater snails. The ostracod assemblage is dominated by *C. torosa* (77-91%) and *Ilyocypris* spp. (7-20%) with few *Candonopsis* (2-3%) and *Pseudocanona* (1-3%) specimens. Density range from 1160 to 4100 valves for 10g of sediments. Excavation of locus 28205 (-213.97/-214.08 m msl) reveals the presence of a piece of wood in the recess of the western wall (Fig. 3 and Fig. S3). This wood, likely used as a "cork", has been dated to 1410 – 1520 years BP (430 – 540 cal. years CE). Locus 28203 is dated to 750 – 910 years BP (1040 – 1200 cal. years CE) and 28202 to 800 – 920 years BP (1030 – 1150 cal. years CE).
These loci are interpreted as a natural infilling of the structure after lake level rise and indicate higher lake levels at that time. The cork is dated from the same period as the structure. We can consider it as a lake limiting point (Fig. 3).

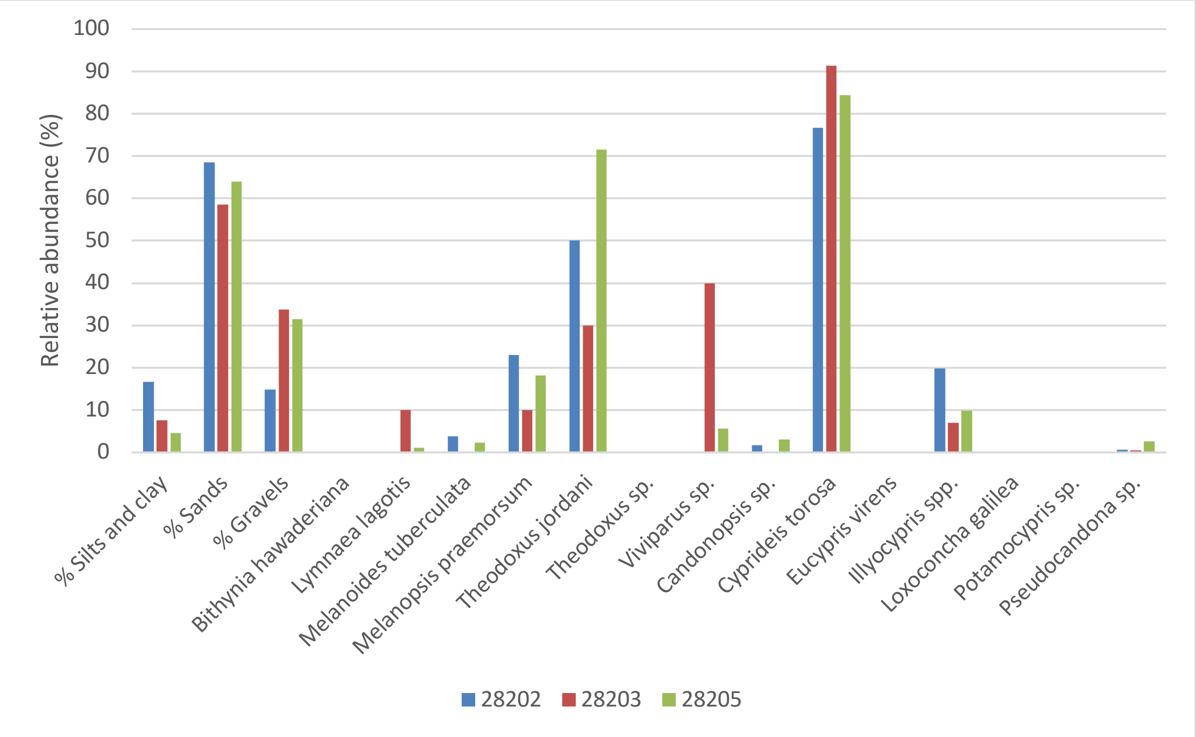

Figure : Sediment texture and fauna for locus 28202, 28203 and 28205

<u>*Loci 28107, 28106, 28105, 28104 and 28102*</u>
A test pit was excavated on the outskirts of the structure to establish the living level of the period of construction of the structure. A similar picture to that of the inner structure was discerned. Seven loci have been excavated.

Locus 28107 is a 10cm thick compact slanting unit, whose bottom is situated between -213.73 and -213.56 cm msl. It is composed of sands (49%) and gravels (30%) with finer sediments (silts and clay=21%). In this locus, we identified *Theodoxus jordani* and *Melanopsis praemorsum*

representing respectively 42% and 29% of the total assemblage. The third species, counting for 19% of the assemblage (n=19) is *Bithynia hawaderiana* living under stones and commonly found along with *T. jordani* and *M. praemorsum* on the shore of the lake (Pollingher et al., 1978; Dzikowski et al., 2003). No ostracods have been found. This locus is dated to the 5-6th century CE (1595 ± 15; 420 – 540 cal. years CE) as the structure (1480 ± 40 BP; 480 – 650 cal. years CE; Galili et al., 2007).
This locus is interpreted as a floor surface containing anthropogenic inputs (grape seeds and pottery). It can be compared with locus 28010 excavated inside the structure but few tens of cm higher and with more coarse sediments and shells originating from the lakeshore. The bottom of this locus is a terrestrial limiting point.

Loci 28106, 28105 and 28104 are located between -213.47 and -213.73 m msl. The samples contain little silts and clay (3%), 51-95% of sands and 2-46 % of gravels. In these samples, *T. jordani* is the dominant mollusc specie (63%), followed by *M. praemorsum* (25%). In locus 28105 – the sandier sample – the air-breathing aquatic snail *Lymnaea lagotis* is also found (20% of the shells discovered in this locus). The ostracod assemblage is dominated by *C. torosa* (82%) and *Ilyocypris* spp. (13%). Density is higher for locus 28106 (1600 valves for 10g of sediments) than for the other two loci (230-340 valves for 10g of sediments). A piece of wood found in locus 28105 has been radiocarbon dated to 920 – 960 years BP (990 – 1030 cal. years CE).
These loci are interpreted as the deposition of shallow coastal sediment near the structure during a period of rising sea level. Not permanently inundated.

Locus 28102 is a pure tight yellow silty sand unit. Silts and clay represents 55% of the total sedimentary texture while the remaining 45% are sands. This sample contains no molluscs. However, the density of ostracods is high (2600 valves for 10 g of sediments). The ostracods assemblage is dominated by *C. torosa* but it represents less than 50% of the total assemblages. *Ilyocypris* spp. (27%) and *Loxoconcha galilea* (26%) are the two other main species of the assemblage.
This locus is also interpreted as the deposition of shallow coastal sediment. It may indicate further rise in sea level. The ostracod fauna is typical of the littoral zone of the lake (Serruya, 1978).

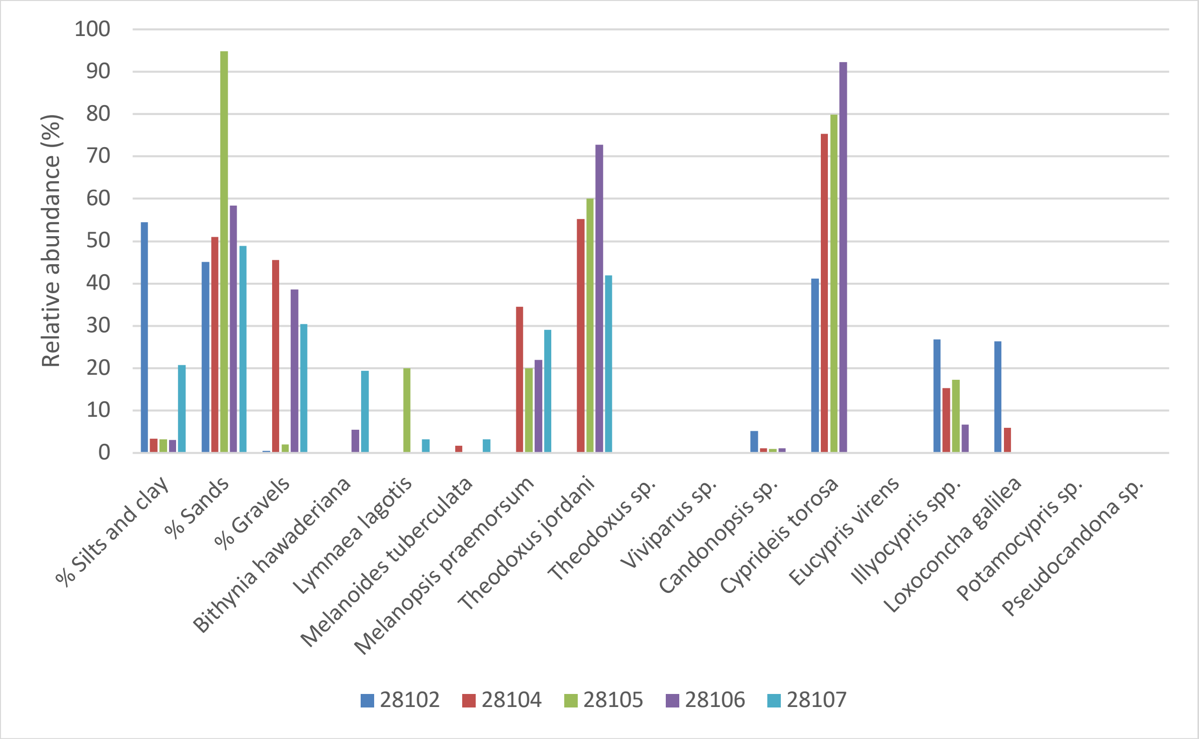

Figure : Sediment texture and fauna for locus 28102, 28104, 28105, 28106, 28107

Lake level index points

Data used to produce the lake level index points represented in Figure 5

ID in Figure 5	Site	Details	Elevation	Dating	Reference
A1	Kursi/Breakwater	Base of the breakwater	-213.40	Iron Age II (1000-800 BCE)	This study
A2	Kursi/Breakwater	Base of the harbor unit	-212.90	Iron Age II (1000-800 BCE)	This study
A3	Kursi/Breakwater	Top of the harbor unit	-212.10	Iron Age II (1000-800 BCE)	This study
A4	Kursi/Breakwater	Top of the breakwater at the time of excavation	-210	Iron Age II (1000-800 BCE)	This study
A5	Kursi/Breakwater	Estimated elevation of the breakwater in the Iron Age II	-209	Iron Age II (1000-800 BCE)	This study
B1	Magdala	Bottom of the harbor basin	-210.2	169 BCE – 4 CE	Sarti et al., (2013)
B2	Magdala	Late-Hellenistic mooring stone	-208.10/-208.17	167 – 63 BCE	Sarti et al., (2013)

B3	Magdala	Early Roman mooring stone	-208.32/-208.23	63 BCE – 1st century CE	Sarti et al., (2013)
B4	Magdala	Late Roman landing zone	-210	Late 3rd century CE	Sarti et al., (2013)
C1	Kursi/Fishpond	Locus 28011	-214.98	80 – 210 CE	This study
C2	Kursi/Fishpond	Cork	-214.08	430 – 540 CE	This study
C3	Kursi/Fishpond	Circulation surface	-213.73	420 – 540 CE	This study
C4	Kursi/Fishpond	Structure elevation	-213.24	480 – 650 CE	Galili et al., (2007)
C5	Kursi/Fishpond	Infilling (crusader period)	-213.95	1030 – 1150 CE	This study
C6	Kursi/Fishpond	Structure elevation	-213.24	1030 – 1150 CE	Galili et al., (2007)

Fig. S1

The breackwater and Tel Kursi (Area A). This image highlight the connection between the breackwater and the tell and reinforce the fact that both of them can be reasonably dated from the Iron Age II period. Photo: Michal Artzy.

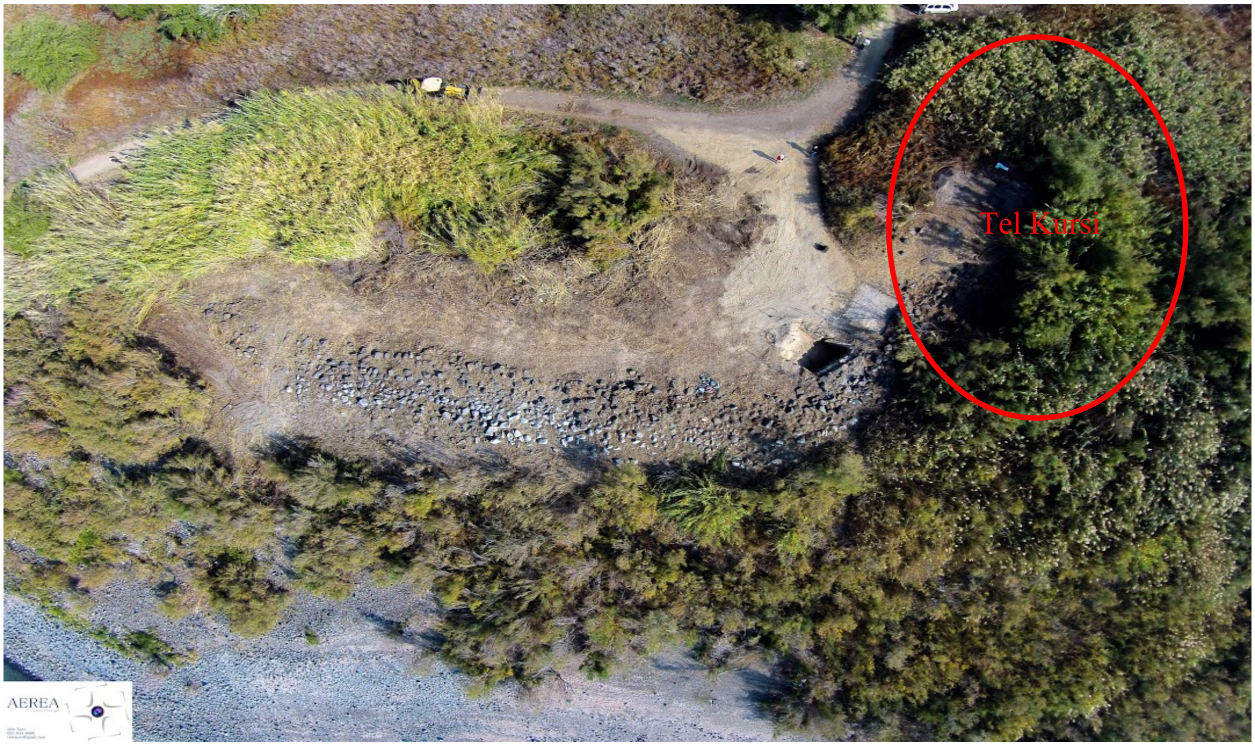

Fig. S2

The fishpond/wishing well excavation (Area C). (a) Plan of Area C/Beach area (M. Edelcopp and B. Arubas); (b) Spillage on the N side of Round Structure (view from the north); (c) Inner and outer excavation of round structure (view from the north-west); (d) Two layers of construction and foundation (view from the west). Photos: Michal Artzy.

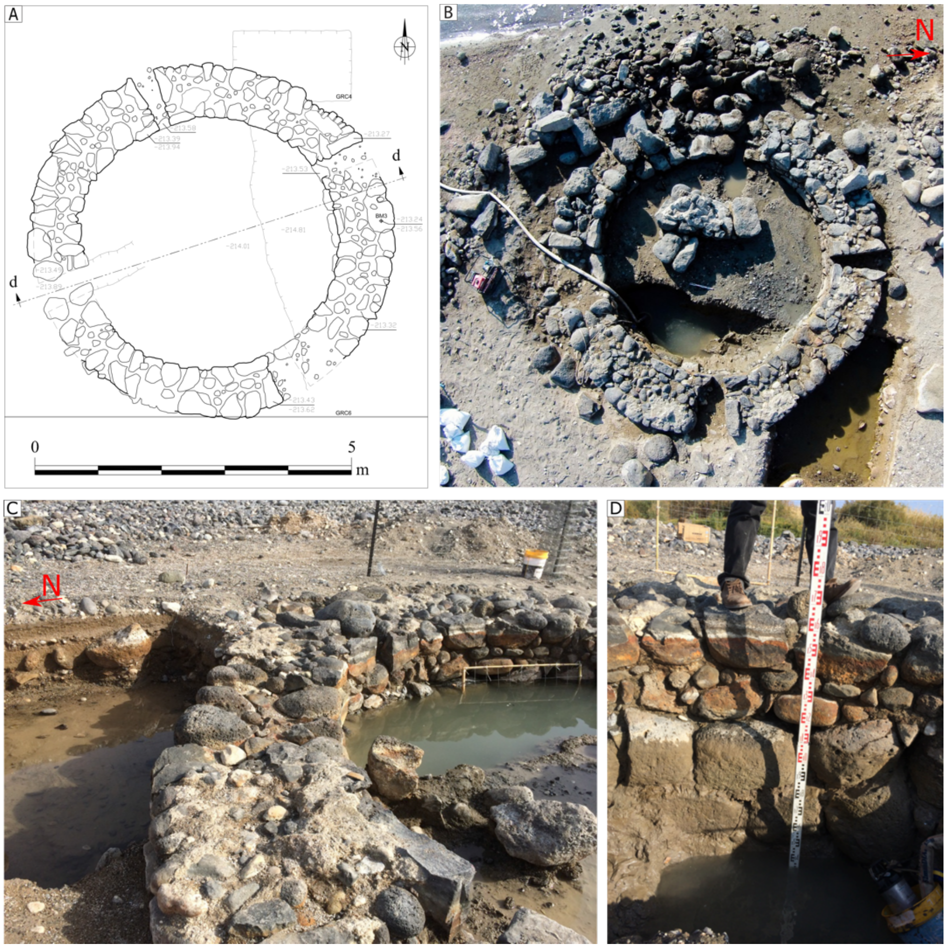

Fig. S3

Lake-level index points obtained from the study of the fishpond at Kursi (Area C) and details of the loci excavated. Description of the loci is given in Supplementary text.

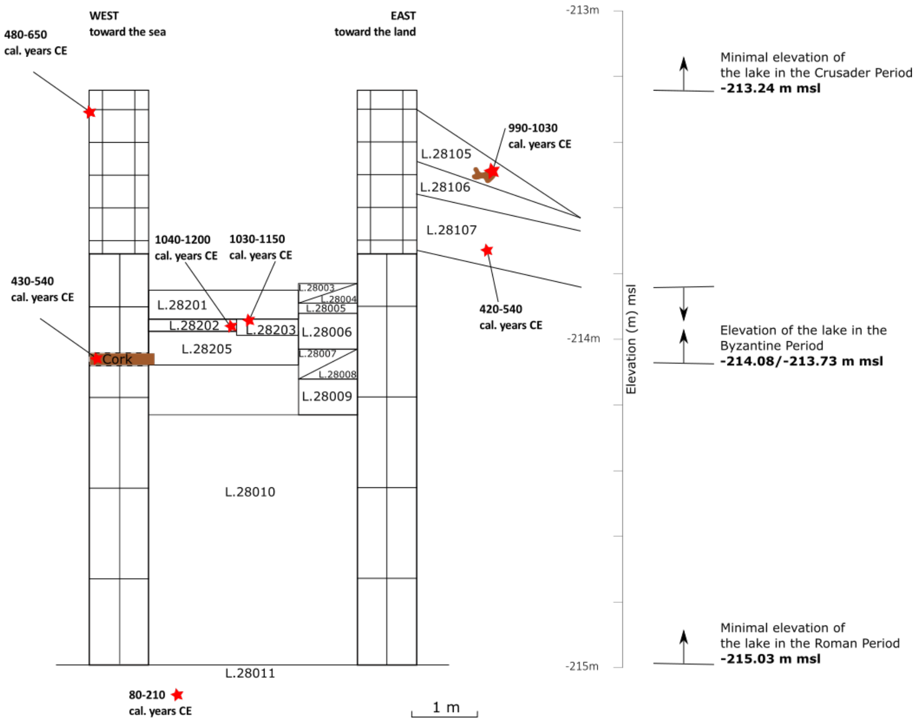

Fig. S4

Wooden log 'cork' in situ, western spillage of round structure (Photo: Michal Artzy)

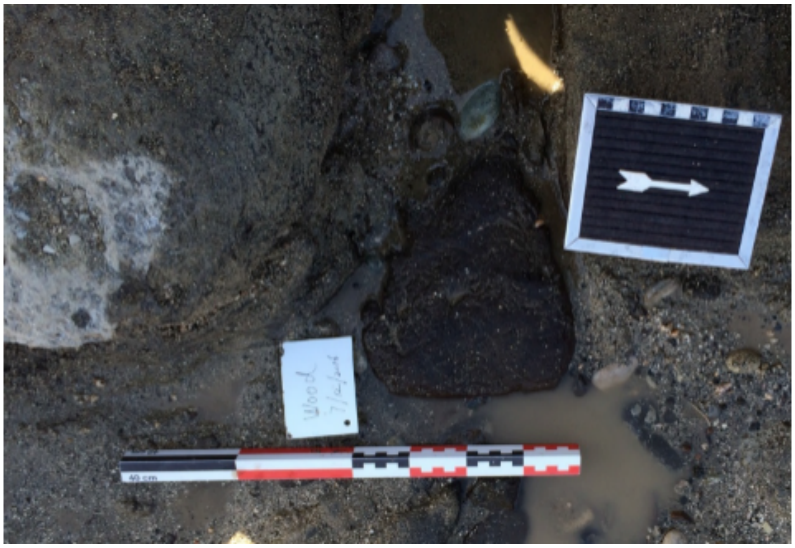

REFERENCES

Dzikowski, R., Diamant, A., Paperna, I. (2003). Trematode metacercariae of fishes as sentinels for a changing limnological environment. Diseases of aquatic organisms 55(2): 145–150.

Galili, E., Rosen, B., Boaretto, E., Tzatzkin, S. (2007). Kursi Beach. Hadashot Arkheologiyot: Excavations and Surveys in Israel/119 חדשות ארכיאולוגיות: חפירות וסקרים בישראל: online.

Meish, C. (2000). Freshwater Ostracoda of western and central Europe. Crustacea.

Mischke, S., Ginat, H., Al-Saqarat, B., Almogi-Labin, A. (2012). Ostracods from water bodies in hyperarid Israel and Jordan as habitat and water chemistry indicators. Ecological Indicators 14(1): 87–99.

Pollingher U., Serruya C., Tsurnamal M., Kugler J., Margalit J., Tahori A.S., (1978). The benthic fauna. In: Serruya C. (eds) Lake Kinneret. Monographiae Biologicae 32. Springer, Dordrecht.

Sarti, G., Rossi, V., Amorosi, A., De Luca, S., Lena, A., Morhange, C., Ribolini, A., Sammartino, I., Bertoni, D., Zanchetta, G. (2013). Magdala harbour sedimentation (Sea of Galilee, Israel), from natural to anthropogenic control. Quaternary International 303: 120–131.

Serruya, C. (1978). The origin of the Kinneret fauna. In C. Serruya (ed), Lake Kinneret, 465–473. Monographiae Biologicae, vol 32. Springer, Dordrecht.
